# Functional Effects of Cyclization on Li1 Peptide Activity against Metacyclic *Leishmania amazonensis* Internalization

**DOI:** 10.1021/acsomega.5c11292

**Published:** 2026-03-02

**Authors:** Túlio Custódio Reis, Ana Clara Lunardi Yagi, Angela Maria Arenas Velásquez, Ana Laura Dias Ramos, Natália Caroline Costa Coelho, Eduardo Maffud Cilli, Márcia A. S. Graminha

**Affiliations:** † Department of Clinical Analysis, School of Pharmaceutical Science, 28108 São Paulo State University (UNESP), Araraquara 14800-903, Brazil; ‡ Latin American Institute of Life and Natural Sciences, Federal University of Latin American Integration (UNILA), Foz do Iguaçu 85870-650, Brazil; § Department of Biochemistry and Chemical Technology, Institute of Chemistry, São Paulo State University (UNESP), Araraquara 14800-060, Brazil

## Abstract

Leishmaniases are caused by protozoa of the genus *Leishmania*, whose metacyclic promastigote forms initiate infection in the mammalian host. Building upon previous work with *Leishmania infantum*, this study evaluated the cyclic peptide Li1 and its linear analogue Li1nc regarding their capacity to interfere with *Leishmania amazonensis* infection. Both peptides were synthesized by solid-phase peptide synthesis (SPPS–Fmoc) and tested after pre-exposure of metacyclic promastigotes prior to infection of murine peritoneal and THP-1–derived macrophages. In contrast to the previous study conducted by our group, which evaluated only Li1 against *L. infantum*, the present work introduces the first direct comparison between the cyclic peptide and its linear analogue, allowing a structure–activity assessment not previously available. Pre-exposure to Li1 (0.5 mg mL^–1^ ≅ 0.318 mmol L^–1^) significantly reduced the infection rate and infection index in both macrophage models, while Li1nc (0.324 mmol L^–1^) exhibited moderate inhibition. Neither peptide displayed cytotoxicity toward host cells (CC_50_ > 0.5 mg mL^–1^) nor direct antipromastigote activity (IC_50_ > 0.5 mg mL^–1^). Confocal microscopy revealed stronger and more defined binding of Li1 to the parasite surface than Li1nc, particularly along the flagellum, supporting structure-dependent interaction with surface molecules involved in parasite internalization. In a murine model of cutaneous leishmaniasis, preincubation of metacyclic promastigotes with Li1 (0.2 mg mL^–1^ ≅ 0.127 mmol L^–1^) reduced parasite burden by 35.2%, whereas Li1nc (0.130 mmol L^–1^) achieved a 20.4% reduction relative to untreated controls. No significant alterations in hepatic or renal biochemical parameters were observed, indicating the absence of systemic toxicity. Notably, neither peptide showed activity under a postinfection treatment regimen (2 mg Kg^–1^), suggesting that their effects are restricted to early host–parasite interactions rather than therapeutic clearance. Collectively, these findings demonstrate that Li1 acts through a structure-dependent mechanism that interferes with host–parasite recognition and reduces infectivity without inducing detectable toxicity. The results support the translational potential of Li1 as a safe peptide scaffold for prophylactic or paratransgenic strategies aimed at preventing *Leishmania* transmission.

## Graphical abstract

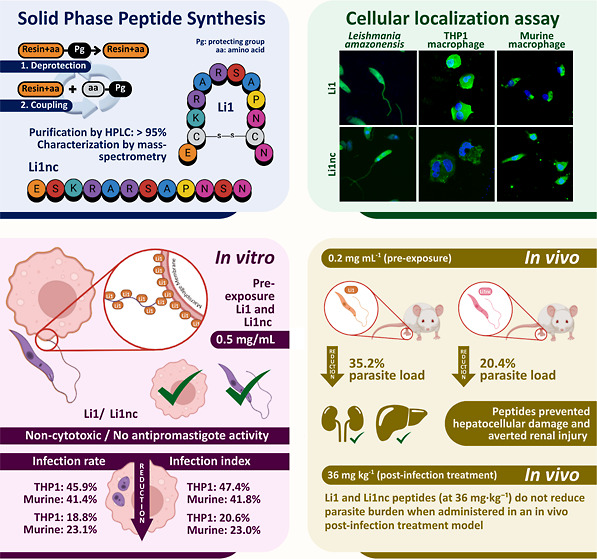

## Introduction

Leishmaniases, caused by protozoa of the genus *Leishmania* (Euglenozoa: Trypanosomatidae), remain priority neglected tropical diseases, with estimated 700,000–1,000,000 new cases annually.^1^ Cutaneous leishmaniasis (CL) is the most prevalent clinical form and is endemic across ∼90 countries, with a substantial and expanding burden in Latin America, where *Leishmania amazonensis* contributes significantly to morbidity. The clinical spectrum depends on both the infecting species and host immune status.^1,2^

Transmission to mammalian hosts occurs during the blood meal of infected female sand flies, represented by *Phlebotomus* spp. in the Old World and *Lutzomyia* spp. in the New World.^3^ Infectivity depends on metacyclogenesis, a process involving coordinated morphological, biochemical, and genetic changes that transform noninfective procyclic promastigotes into highly virulent metacyclic forms.^4,5^ These metacyclic parasites remodel their surface glycocalyx, becoming enriched in virulence determinants such as lipophosphoglycan (LPG) and leishmanolysin (GP63).^6,7^ Other surface molecules—including proteophosphoglycan (PPG), kinetoplastid membrane protein 11 (KMP-11), glycoprotein 46 (GP46), and hydrophilic acylated surface protein (HASP)–are also up-regulated during metacyclogenesis, facilitating adhesion, immune evasion, and intracellular survival in mononuclear phagocytic cells, primarily macrophages.^8–10^

Despite advances in chemotherapy, treatment options for leishmaniasis remain limited by toxicity, parenteral administration, prolonged regimens, and the emergence of resistant isolates.^11–13^ Consequently, recent research has shifted toward identifying ligands capable of preventing the internalization of *Leishmania* into host cells, rather than developing new therapeutic drugs.^14^ Among these approaches, phage-display peptide libraries have proven effective for selecting ligands with high affinity for parasite-surface molecules, as shown by Rhaiem and Houimel^15^ and by our research group.^14^ In the latter study, a 12-amino-acid cyclic peptide designated Li1 was identified for its ability to bind to the surface of *Leishmania infantum*, the etiological agent of visceral leishmaniasis, the most severe form of the disease, with fatality rates exceeding 90% in untreated cases.^1^ Pre-exposure of *L. infantum* metacyclic promastigotes to Li1 significantly inhibited parasite internalization in macrophages and reduced parasite loads by more than 80% in the spleen and liver of infected BALB/c mice.^14^ Importantly, these findings supported Li1 as a prophylactic candidate aimed at blocking parasite entry, rather than as a therapeutic drug for treating established infections. However, several key questions remained unresolved. First, it was unknown whether this activity extended to *L. amazonensis*, an etiological agent of cutaneous leishmaniasis. Second, the contribution of peptide cyclization to biological activity had never been experimentally tested, as no prior study compared Li1 with its linear analogue Li1nc. Conformationally constrained structures, produced by cyclization, often exhibit remarkable therapeutic properties, such as high binding affinity, specificity and proteolytic stability.^16^ Third, the interaction of Li1 with host macrophages, rather than exclusively with parasites, had not been explored. Finally, the previous work did not examine Li1 in human macrophage models, nor did it assess postinfection therapeutic potential, in vivo biosafety parameters, or structural determinants of surface binding.

To address these gaps, the present study expands on the work of Verga et al.^14^ by evaluating the ability of Li1 and Li1nc to block *L. amazonensis* internalization, using two complementary macrophage models—human THP-1-derived macrophages and murine peritoneal macrophages—and by assessing their prophylactic efficacy in a murine model of cutaneous leishmaniasis, at the same concentration previously tested *in vivo*. By incorporating a direct structural comparison (cyclic versus linear), evaluating peptide binding to both parasite and host cell surfaces, and examining liver and kidney biochemical markers, this study provides new mechanistic and translational insights into peptide-based inhibition of *Leishmania* internalization. These advances significantly extend the original findings and clarify the structural and biological determinants underlying Li1 activity.^16^ Our results demonstrate a structure-dependent inhibitory effect of Li1 on the internalization of metacyclic forms and a corresponding reduction in parasite burden *in vivo*, supporting its application as a prophylactic tool—potentially in vector-blocking or paratransgenic approaches—rather than as a therapeutic drug for established infections.^14^

Because Li1 was selected through phage display against whole metacyclic promastigotes, its cognate ligand remains unknown. Therefore, structural modeling was not applied, and mechanistic interpretations are limited to experimentally observed differences between cyclic and linear analogues (Figure 1).

**Figure 1:**
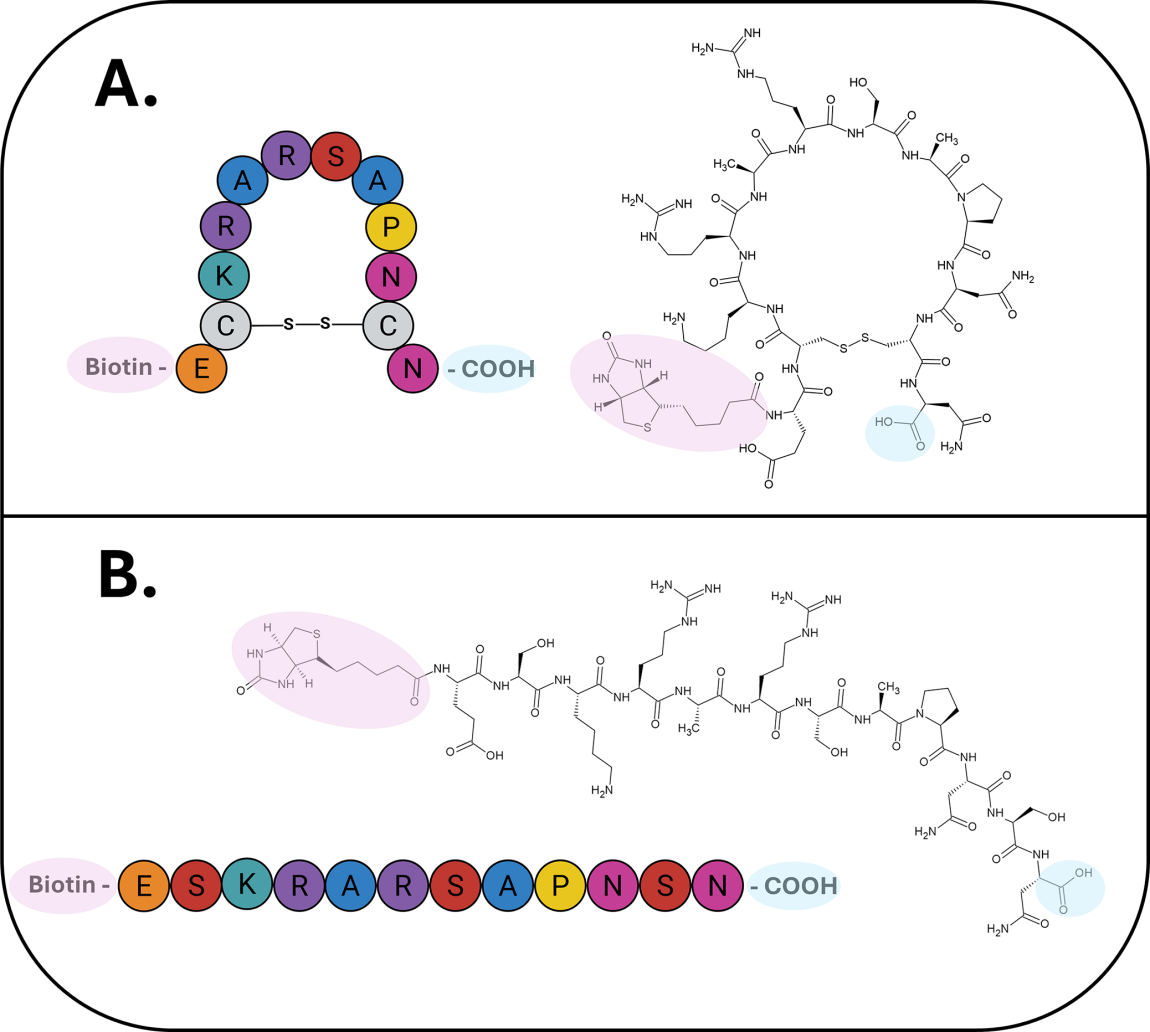
Schematic representation of the amino acid residue sequences of the Li1 and Li1nc peptides, as well as their corresponding chemical structures. (A) Structure of the Li1 (Biotin-EC^|^KRARSAPNC^|^N–COOH) peptide, composed of 12 amino acid residues with cysteines fixed at positions 2 and 11, which form a disulfide bond between their sulfhydryl (-SH) groups. This bond creates an eight-residue loop, conferring a cyclic conformation to the peptide. (B) Structure of the Li1nc (Biotin-ESKRARSAPNSN-COOH) peptide, consisting of 12 amino acid residues with serines fixed at positions 2 and 11, preventing cyclization through disulfide bonding which avoids disulfide-mediated cyclization and results in a linear conformation of the peptide. The chemical structures of both peptides were generated by the author using the ACD/ChemSketch software (Figure 1 was created using BioRender.com).

## Results and Discussion

### Synthesis and Characterization of the Li1 and Li1nc Peptides

Both peptides, Li1 (cyclic) and its linear analogue Li1nc, were synthesized by solid-phase peptide synthesis (SPPS) using Fmoc chemistry under previously optimized conditions for phage-derived peptides.^17^ ^,^ ^18^ The cyclic structure of Li1 was obtained through disulfide bond formation between cysteine residues at positions 2 and 11, whereas these residues were replaced by serines in Li1nc to prevent cyclization. This design (Ser substitution) ensured that Li1nc could not form an intramolecular bridge; however, the Cys → Ser substitution also introduces differences in side-chain chemistry (thiol versus hydroxyl), so Li1 should be interpreted as stable structurally.^19^

Cyclization was achieved by iodine oxidation as described by Reddy et al.^20^ After purification by reverse-phase HPLC, both peptides displayed >95% purity (Figure 2), and ESI-MS analysis confirmed their expected molecular masses (Li1:1572.8 Da; Li1nc: 1542.7 Da) with corresponding *m*/*z* values of 787.08 and 525.17 for Li1, and 772.27 and 515.44 for Li1nc (Figure 3).

**Figure 2:**
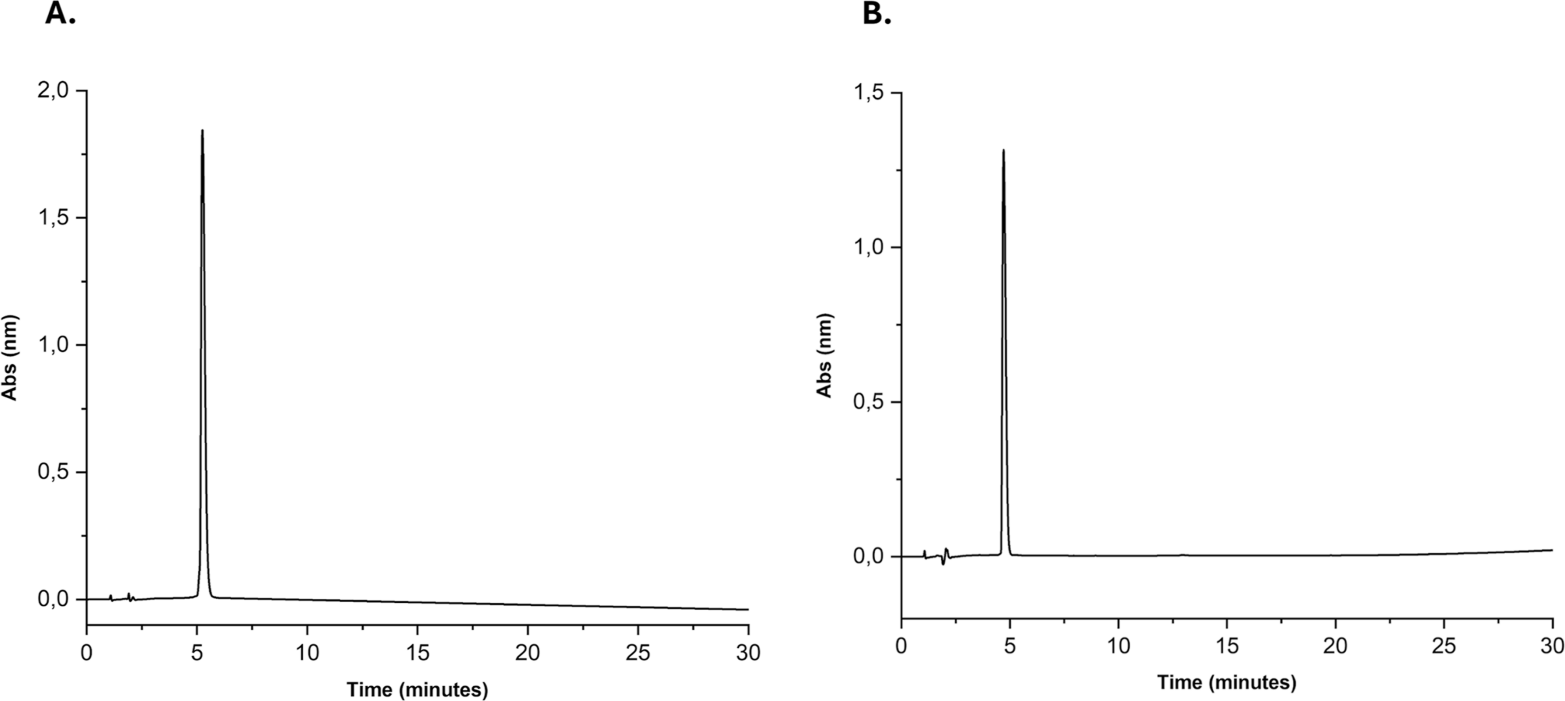
Chromatographic purification profiles of the synthetic peptides Li1 and Li1nc obtained by reverse-phase HPLC. (A) Analytical chromatogram of the cyclic biotinylated peptide Li1 showing a retention time of 5.2 min under a 5–95% solvent B gradient over 30 min (solvent A: 0.045% trifluoroacetic acid (TFA) in water; solvent B: 0.036% TFA in acetonitrile). (B) Analytical chromatogram of the linear biotinylated analogue Li1nc showing a retention time of 4.7 min under identical conditions. Chromatographic separations were performed using a C18 reverse-phase column (25 cm × 10 mm, 5 μm; Jupiter Proteo) with a flow rate of 1 mL min^–1^ and UV detection at 220 nm. Both peptides displayed a single dominant peak corresponding to a purity >95%, confirming successful synthesis and purification suitable for subsequent biological assays.

**Figure 3:**
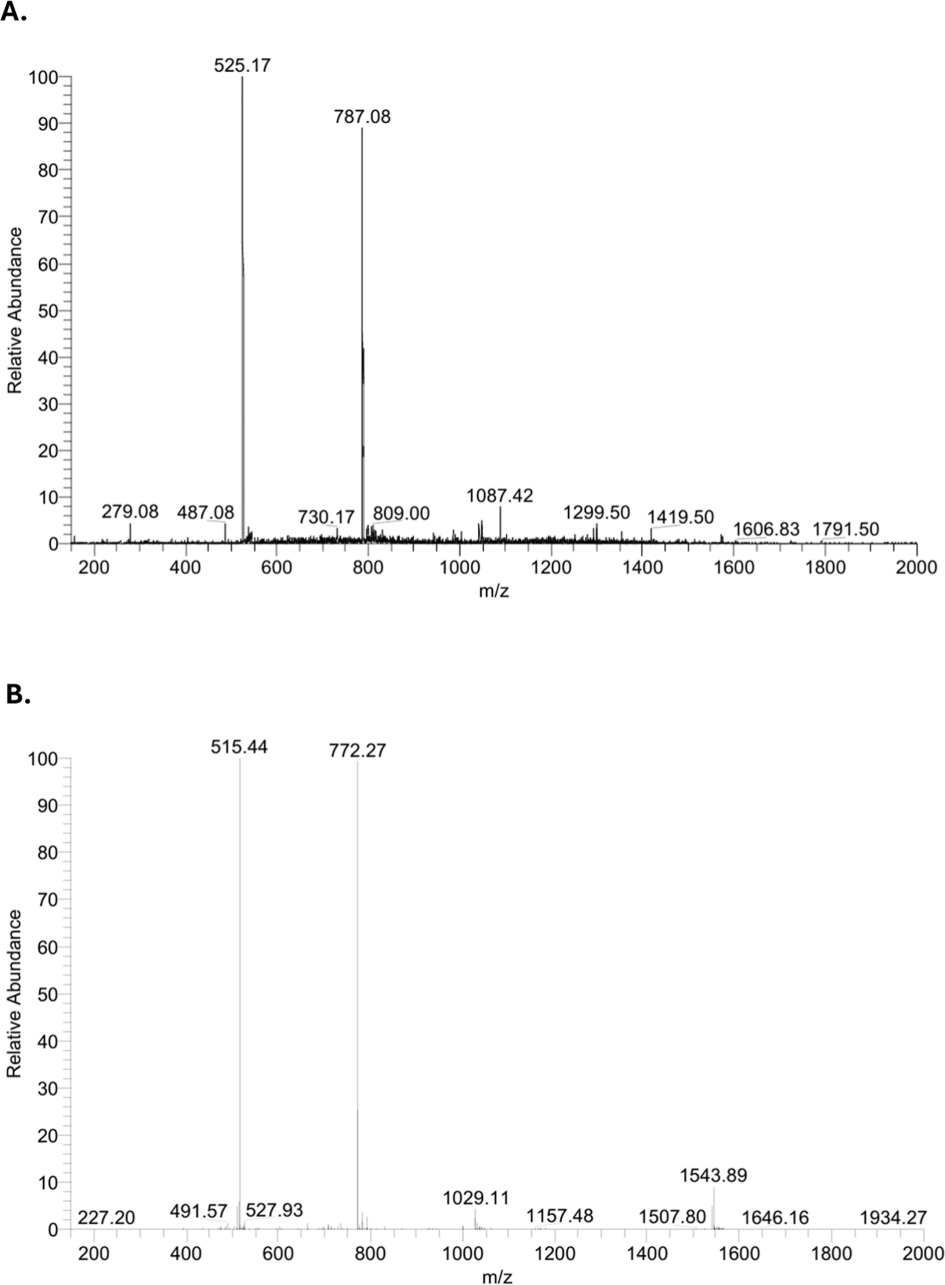
Electrospray ionization–ion trap mass spectra (ESI-IT-MS) confirming the molecular masses of Li1 and Li1nc peptides. (A) Mass spectrum of cyclic Li1 showing peaks at *m*/*z* = 787.08 and 525.17, corresponding to doubly and triply charged ions (Z = 2, 3), with a calculated molecular mass (M^+^) of 1572.83 Da. (B) Mass spectrum of linear Li1nc showing peaks at *m*/*z* = 772.27 and 515.44 for Z = 2 and 3, with a calculated molecular mass (M^+^) of 1542.70 Da. Analyses were performed on an LCQ Fleet (Thermo Fisher Scientific) ion trap mass spectrometer in positive electrospray mode with a detection range of *m*/*z* 200–2000.

A more refined dissection of constrained conformational would benefit from additional analogues through the use of unnatural amino acids, side-chain modification, *N*-alkyl-amino acids, d-amino acids, chemically blocked cysteines and other bridging methodology, which is being considered for future structure–activity studies.^21^

The retention time of Li1 (5.2 min) was slightly longer than that of Li1nc (4.7 min), reflecting its lower polarity and higher conformational rigidity due to disulfide bond formation (Figure 2). This conformational constraint, as reported for cyclic peptides, confers enhanced proteolytic stability and favorable residue orientation for target interaction.^19,21^ The overall yields (60% for Li1; 91% for Li1nc) are consistent with the additional oxidation step required for cyclization and align with those reported by Verga et al.^14^

### Pre-Exposure of Metacyclic Forms to Li1 or Li1nc Reduces Parasite Internalization in Macrophage Models

The growth curve of *L. amazonensis* (Figure 4A) showed the onset of the stationary phase on day 5, with the proportion of metacyclic forms peaking on days 6–7 at approximately 40–45% (Figure 4B). Promastigotes from these days were enriched by Ficoll density gradient and used in infection assays, as standardized by Späth & Beverley.^22^ Although the total parasite density decreases between days 6 and 7 (Figure 4A), the percentage of metacyclic promastigotes does not exhibit a corresponding decline because metacyclic constitute only a subpopulation within the culture. During the stationary and early decline phases, this subpopulation remains relatively stable, even as the overall number of promastigotes decreases.

**Figure 4:**
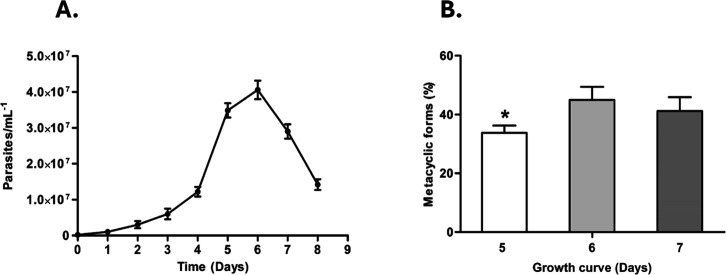
Growth curve and proportion of metacyclic promastigotes of *L. amazonensis*. (A) Growth curve of *L. amazonensis* promastigotes initiated with an inoculum of 2 × 10^5^ parasites mL^–1^ and maintained at 26 °C for 8 days. Parasite density was measured daily using a Neubauer chamber. (B) Percentage of metacyclic promastigote forms on days 5, 6, and 7 of culture, determined by Giemsa staining based on morphological criteria (flagellum length ≥ cell body). Data are expressed as the mean ± standard deviation from three independent experiments. Asterisks (*) indicate statistically significant differences relative to day 6 (*P* < 0.01). The proportion of metacyclic forms increased from day 5 to 6, reaching approximately 40–45% and stabilizing thereafter, defining the optimal time window for enrichment and infection assays.

Infection assays with differentiated THP-1 and murine peritoneal macrophages revealed that pre-exposure of metacyclic forms to the cyclic Li1 peptide (0.5 mg mL^–1^, 15 min) significantly reduced both infection rate and infection index compared with untreated controls (Figure 5A,B). In THP-1 cells, Li1 decreased the infection rate by approximately 45.9% and the infection index by 47.4%, while in murine macrophages the reductions were 41.4% and 41.8%, respectively. The linear analogue Li1nc induced only modest inhibition (≈20%), showing that the rigid structure of the cyclic peptide is important to biological activity.

**Figure 5:**
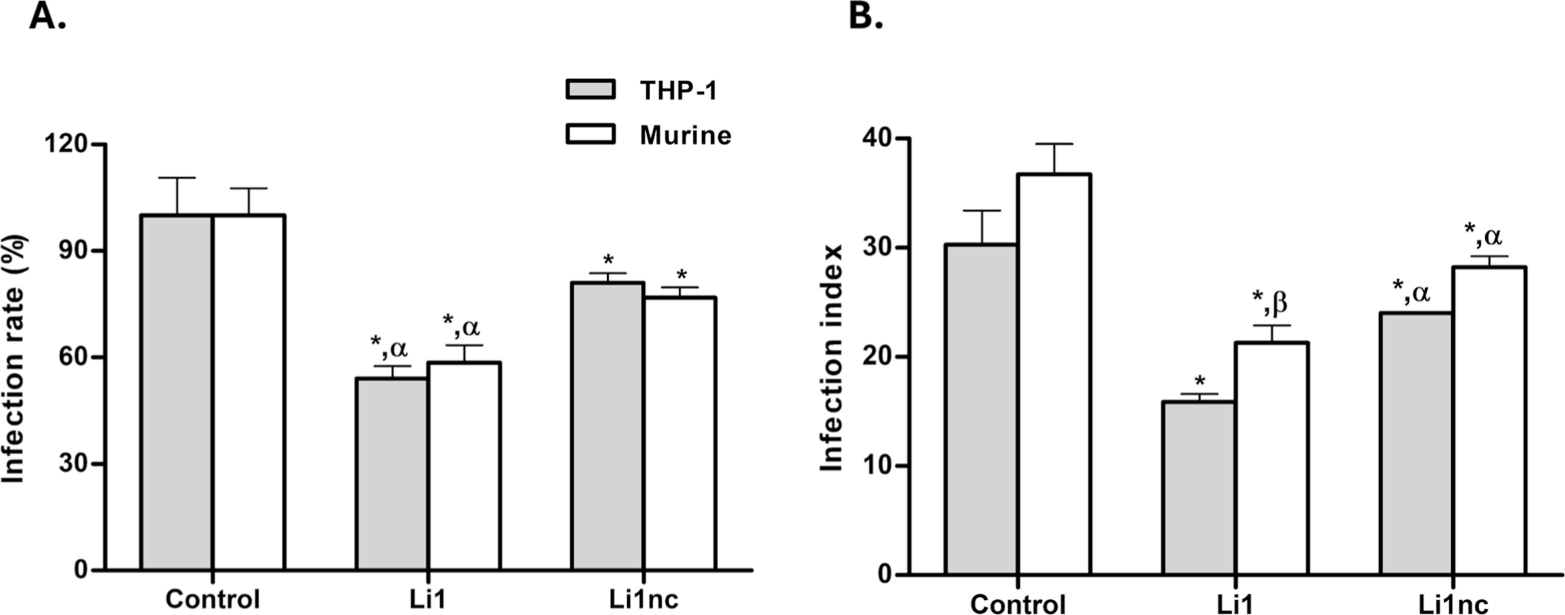
Effect of Li1 and Li1nc peptides on *L. amazonensis* infection in macrophage models. (A) Infection rate (%) of murine peritoneal macrophages and THP-1–derived macrophages infected with *L. amazonensis* metacyclic promastigotes pre-exposed to Li1 or Li1nc peptides (0.5 mg mL^–1^, 15 min) compared with untreated controls (PBS). (B) Infection index, calculated as the mean number of intracellular parasites per 100 macrophages, for the same experimental groups. Bars represent the mean ± standard deviation (*n* = 3 independent assays, each in triplicate). Asterisks (*) denote statistically significant differences compared with the control group (*P* < 0.05); Greek letters indicate pairwise comparisons: α = significant difference between Li1 and Li1nc within the same macrophage model (*P* < 0.001); β = significant difference for the same peptide between macrophage models (*P* < 0.01).

These data confirm a structure-dependent reduction in parasite internalization, paralleling the effects of the parent cyclic peptide Li1 against *L. infantum* reported by Verga et al.^14^ Cyclic peptides generally exhibit improved affinity and selectivity due to their restricted conformational freedom and enhanced stability.^23–25^ In this context, the superior efficacy of Li1 supports an important contribution of conformational constraint to the inhibitory effect.

The superior efficacy of Li1 therefore supports the critical role of cyclization in promoting binding to surface molecules involved in *Leishmania* entry. Because the aim of this study was specifically to determine whether the peptides increased or reduced parasite internalization—rather than to develop or characterize them as therapeutic drugs—only a single concentration was used. This approach allowed a direct mechanistic comparison between Li1 and Li1nc. Accordingly, the present data establish inhibitory activity at this dose but do not define a dose response relationship or EC_50_ values, which should be addressed in future investigations to further advance the discussion of the present study and to provide definitive evidence of the intrinsic potential of the Li1 peptide.

### Li1 and Li1nc Display No Cytotoxic or Direct Antiparasitic Effects

To determine whether the reduced infection rates resulted from cytotoxicity, both peptides were evaluated against stationary-phase promastigotes and macrophage cultures. These data were obtained from MTT assays performed in triplicate and are presented as cell viability percentages (%) (Table S1, Supporting Information). The IC_50_ and CC_50_ values were >0.5 mg/mL for both peptides, indicating no direct antipromastigote or cytotoxic effects at the concentrations used in the infection assays. Amphotericin B, included as a positive control, displayed an IC_50_ of 0.6 μg mL^–1^, validating the assay.

Thus, the observed reduction in infection rate and index arises from interference with host–parasite interactions rather than cellular toxicity, corroborating the findings of Verga et al.^14^

### Confocal Microscopy Reveals Peptide Binding to Parasite and Host Cell Surfaces

To assess whether the peptides interact with the surface of *L. amazonensis* metacyclic forms, biotinylated Li1 and Li1nc were visualized by confocal fluorescence microscopy after incubation with Alexa Fluor 488–conjugated streptavidin. Both peptides produced peripheral fluorescence outlining the parasite body and flagellum, consistent with surface-associated labeling under the conditions tested.

However, the fluorescence patterns differed markedly between the two peptides. The cyclic peptide Li1 generated a stronger and more continuous signal, clearly delineating the parasite contours with high intensity, whereas Li1nc produced weaker and more diffuse fluorescence (Figure 6A). The control group (PBS + streptavidin-488) showed no detectable labeling, confirming the specificity of detection for the biotinylated peptides.

**Figure 6:**
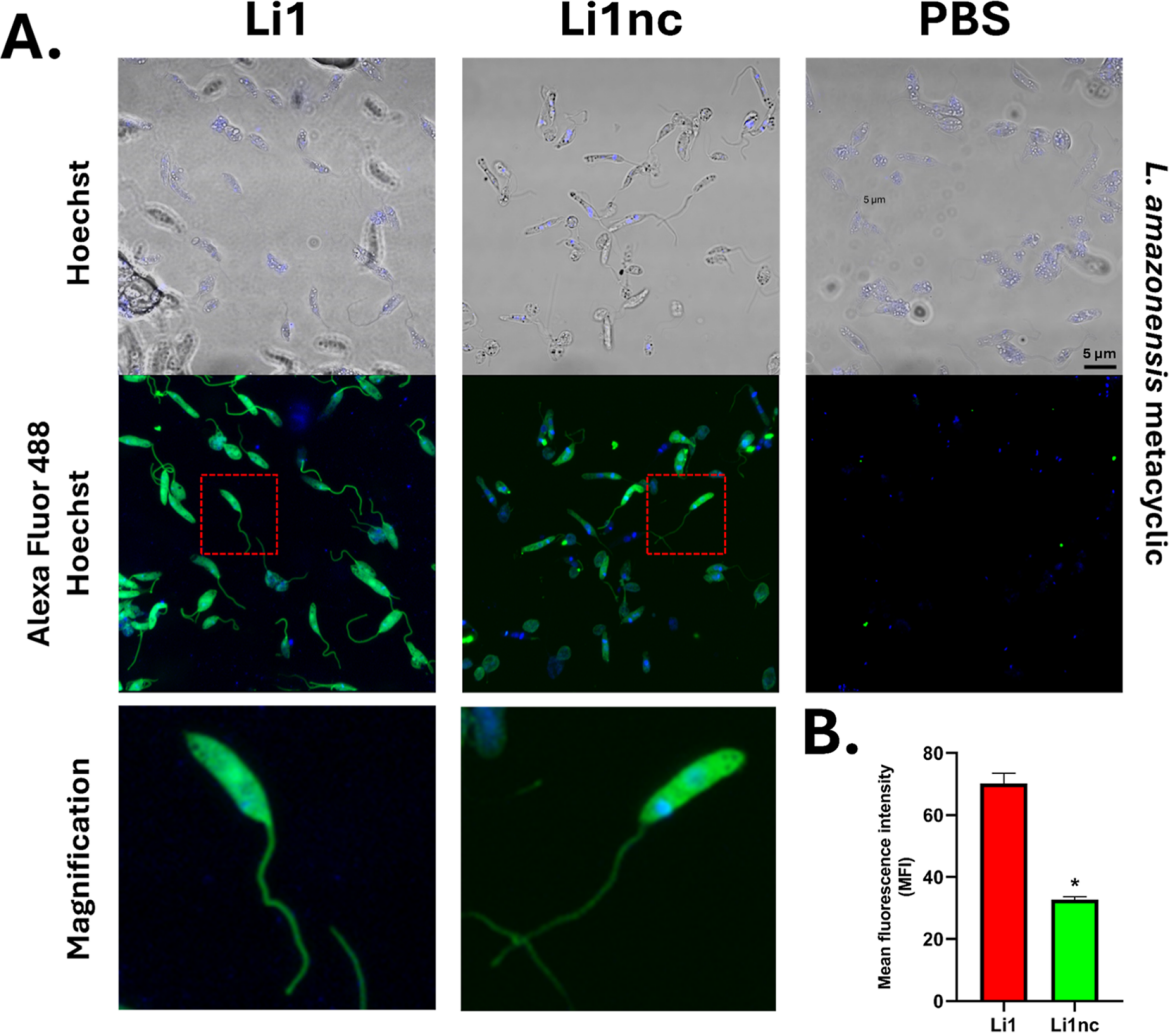
Confocal fluorescence microscopy and quantitative analysis of Li1 and Li1nc binding to the surface of *L. amazonensis* metacyclic promastigotes. (A) Metacyclic promastigotes (1 × 10^5^) were fixed with 4% (v/v) paraformaldehyde and incubated with biotinylated Li1 or Li1nc peptides (0.5 mg mL^–1^) for 8 h at room temperature. Bound peptides were visualized using Alexa Fluor 488–conjugated streptavidin (green), and nuclear DNA was counterstained with Hoechst 33342 (blue). Images were acquired on a Zeiss LSM800 confocal microscope under identical laser and gain settings. Representative images show peripheral fluorescence outlining the parasite body and flagellum for both peptides, with higher signal intensity for Li1. (B) Quantification of mean fluorescence intensity (MFI) by analyzing regions of interest (ROI = 50), along the parasite surface was performed using Fiji (ImageJ) with background subtraction. Bars represent mean ± standard deviation from multiple fields across independent experiments. Statistical comparisons were performed using an unpaired two-tailed Student’s *t*-test; *P* < 0.05 was considered statistically significant (*) compared with Li1. No fluorescence was detected in the control group (PBS + streptavidin–Alexa 488), confirming labeling specificity.

Supporting these qualitative observations, quantification of mean fluorescence intensity (MFI) revealed a significantly higher fluorescence signal on the surface of metacyclic forms incubated with Li1 (Figure 6B), indicating that Li1 produces a stronger and more defined signal along the parasite contour compared with its linear analogue. This finding is consistent with previous results for the parental cyclic peptide Li1 against *L. infantum*, in which cyclization enhanced peptide stability and target-binding capacity.^14^

Based on the observations presented here, on preliminary cellular localization experiments and affinity-based proteomic pull-down assays, studies currently being conducted with metacyclic and procyclic forms by our research group detected a greater interaction with surface-isolated proteins from metacyclic forms (data not shown). This may suggest that the surface ligand that interact with Li1 peptide is present in both forms but more highly expressed in metacyclic promastigotes - raising the possibility that the peptide may interact with a single surface ligand upregulated in the metacyclic form or that Li1 interacts with multiple ligands present in both developmental stages.^14^ However, we cannot identify a specific surface molecule based on the present data.

Moreover, the distribution of Li1-associated fluorescence along the flagellum may indicate association with surface-exposed components involved in parasite adhesion and internalization, which are upregulated during metacyclogenesis.^10,25,26^ However, because the present imaging approach cannot resolve whether the peptide remains exclusively on the parasite surface or crosses the membrane, partial internalization cannot be confirmed or ruled out. Future colocalization assays using intracellular or membrane markers will be important to validate the possibility of peptide internalization. Beyond the qualitative observation of a consistent difference in fluorescence across replicates, the quantitative analysis and the significant MFI increase further support the hypothesis that the cyclic conformation contributes to stronger fluorescence labeling under identical conditions. It is important to note, however, that in the present study we did not include a cyclic scrambled Li1 analogue as a control. In Verga et al.,^14^ a cyclic scrambled peptide (Li1scr, sequence: ACRKNRESNACP) with identical amino acid composition but randomized sequence showed no activity *in vitro* or *in vivo*, reinforcing the importance of primary sequence in addition to conformational constraint. In the absence of a scrambled cyclic control here, our mechanistic interpretation remains conservative and focuses on structure-dependent effects rather than specific molecular targets.

To examine whether these peptides also interact with host cells, THP-1–derived macrophages and murine peritoneal macrophages were incubated with the biotinylated peptides under identical conditions. Both peptides displayed membrane-associated fluorescence, consistent with surface labeling, although the current 2D imaging cannot fully distinguish surface-bound signal from potential internalization (Figures 7A and 8A). MFI quantification showed higher fluorescence on the surface of THP-1 macrophages exposed to Li1 (Figure 7B), with significant differences relative to Li1nc. Interestingly, despite qualitative differences in signal distribution, both peptides showed high fluorescence in the murine macrophage model (Figure 8B), with no significant differences between them.

**Figure 7:**
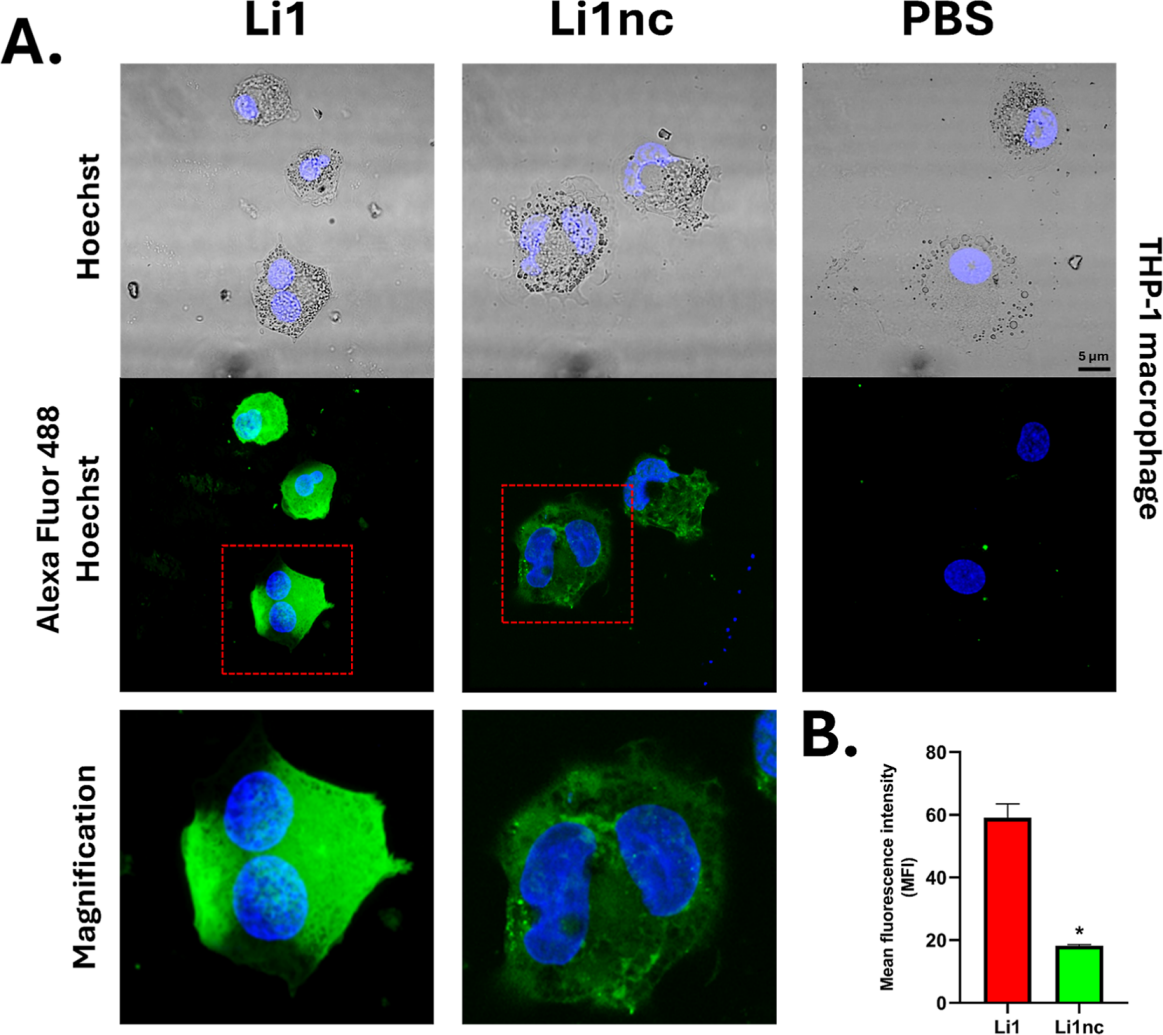
Confocal fluorescence microscopy and quantitative analysis of Li1 and Li1nc binding to THP-1 macrophage surfaces. (A) THP-1 macrophages were fixed with 4% (v/v) paraformaldehyde and incubated with biotinylated Li1 or Li1nc peptides (0.5 mg mL^–1^, 8 h, room temperature). Peptide binding was visualized using Alexa Fluor 488–conjugated streptavidin (green), and nuclei were stained with Hoechst 33342 (blue). Images were acquired under identical confocal settings. (B) Quantification of mean fluorescence intensity (MFI) by analyzing regions of interest (ROI = 30), along the macrophage plasma membrane. Values represent mean ± standard deviation from multiple fields across independent experiments. Statistical analysis was performed using an unpaired two-tailed Student’s *t*-test, and *P* < 0.05 was considered statistically significant (*) compared with Li1. The control group (PBS + streptavidin–Alexa 488) showed no detectable fluorescence.

**Figure 8:**
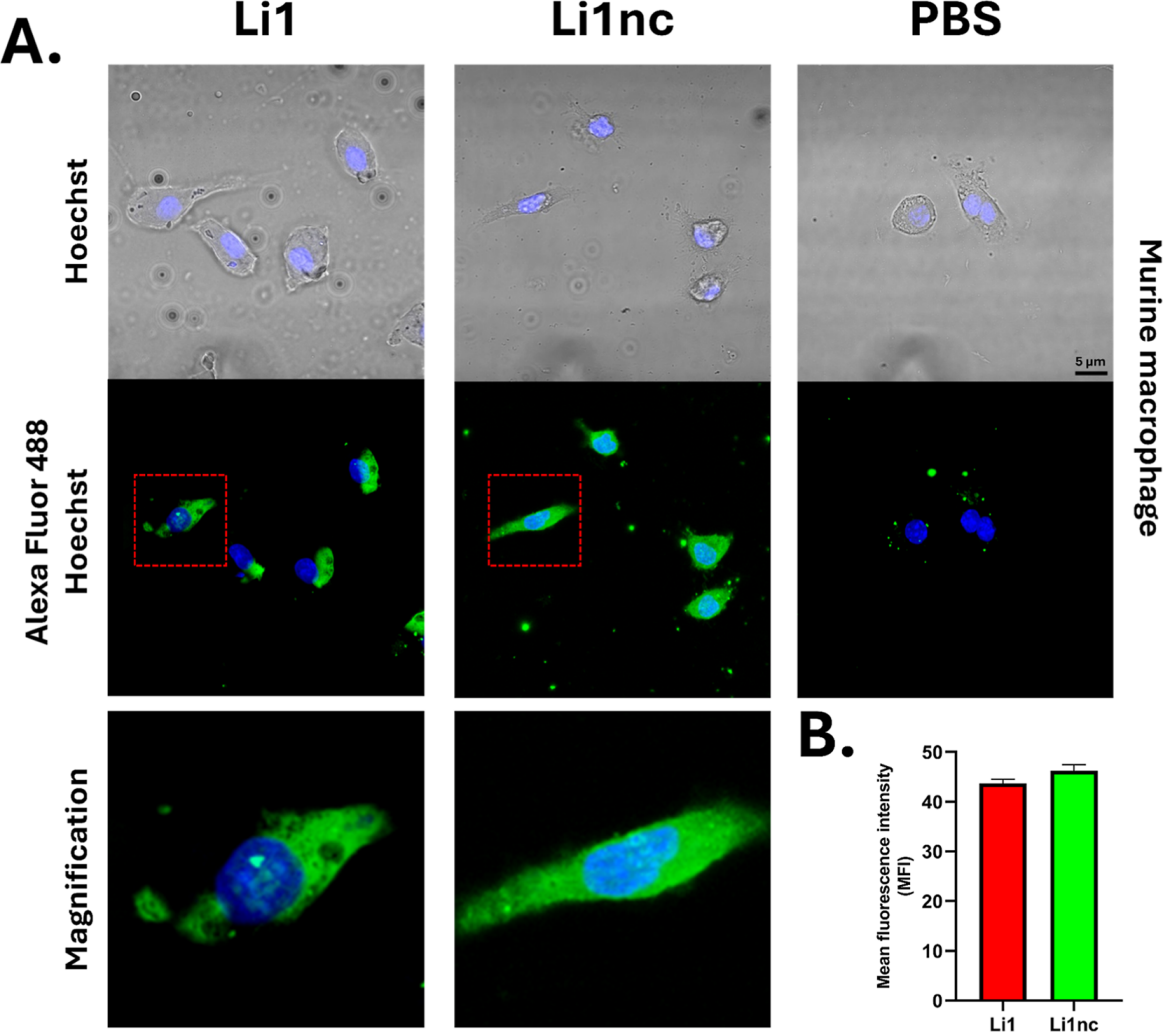
Confocal fluorescence microscopy and quantitative analysis of Li1 and Li1nc binding to murine peritoneal macrophage surfaces. (A) Murine peritoneal macrophages were fixed with 4% (v/v) paraformaldehyde and incubated with biotinylated Li1 or Li1nc peptides (0.5 mg mL^–1^, 8 h, room temperature). Alexa Fluor 488–conjugated streptavidin was used for detection (green), and nuclei were stained with Hoechst 33342 (blue). (B) Quantification of mean fluorescence intensity (MFI) by analyzing regions of interest (ROI = 30), along the macrophage surface. Values represent mean ± standard deviation from multiple fields across independent experiments. No statistically significant differences were observed between Li1 and Li1nc (*P* > 0.05), as determined by an unpaired two-tailed Student’s *t*-test. No fluorescence signal was detected in the control group (PBS + streptavidin–Alexa 488).

This represents a mechanistic advance relative to Verga et al.,^14^ as the previous study did not examine peptide interaction with host macrophages. By demonstrating that Li1 also binds to macrophage surfaces, the present work provides the first indication that the peptide may influence the early parasite–host interface rather than acting solely through parasite recognition. This pattern suggests that Li1 may interact with complementary molecular partners on both the parasite and the host cell, thereby interfering with adhesion and internalization processes.

Although the exact targets remain to be elucidated and additional conformational controls would be valuable, the strong host-cell surface binding evidenced by Li1 fluorescence is consistent with its greater inhibitory effect observed in the *in vitro* infection assays and *in vivo* experiments.

### Li1 Reduces Parasite Burden and Lesion Development *In Vivo* in a Prophylactic Treatment Model

After 30 days of infection, parasite burden in paw tissues was quantified using the limiting dilution method (Figure 9A). Mice infected with *L. amazonensis* metacyclic promastigotes pre-exposed to the cyclic Li1 peptide (0.2 mg mL^–1^, 15 min) exhibited a 35.2% reduction in parasite load compared with the untreated control. The linear analogue, Li1nc, also produced a statistically significant, though less pronounced, reduction of 20.4%. Both Li1 and Li1nc treatments led to visibly reduced paw swelling relative to the control group (Figure 9B).

**Figure 9:**
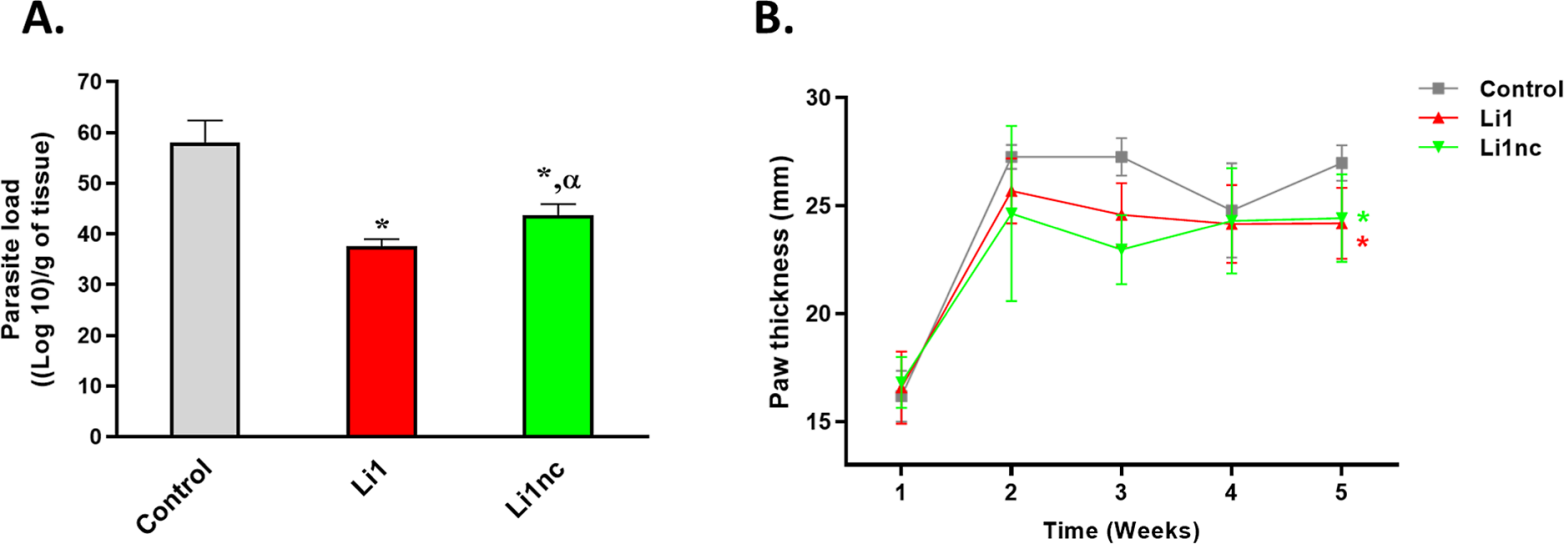
Parasite burden and lesion development in BALB/c mice infected with *L. amazonensis* metacyclic promastigotes preincubated with Li1 or Li1nc peptides. (A) The parasite load was determined by the limiting dilution method 30 days postinfection and (B) paw thickness was measured using a digital caliper every week postinfection. Metacyclic promastigotes from day 6 of culture were pre-exposed to Li1 or Li1nc peptides (0.2 mg mL^–1^, 15 min) prior to inoculation into the right hind paw of BALB/c mice (*n* = 5 per group). Data are expressed as mean ± standard deviation. Statistical analysis was performed by One-way ANOVA followed by Tukey’s post hoc test (*P* < 0.05). Asterisks (*) denote statistically significant differences compared with the control group (*P* < 0.0001); Greek letters indicate pairwise comparisons: α = significant difference between Li1 and Li1nc (*P* < 0.05).

This pattern indicates that both peptides attenuate lesion development primarily by impairing the initial parasite–host interaction and modulating local inflammation, rather than by affecting parasite replication. Importantly, the more pronounced reduction in parasite burden observed with the cyclic Li1 peptide suggests that structural features associated with cyclization, such as enhanced stability and restricted conformational freedom, improve its capacity to interfere with parasite adhesion or recognition at the host cell surface. Given the Cys → Ser substitution in Li1nc, these differences likely reflect a combination of conformational and side-chain effects and cannot be assigned to cyclization alone.

Together, these results corroborate the inhibitory effects observed *in vitro* and support a structure-dependent mechanism in which the cyclic conformation of Li1 confers superior biological activity by disrupting parasite–host interactions. The observed prophylactic effect further reinforces Li1 as a promising scaffold for infection-blocking strategies, extending previous findings from *L. infantum* to the cutaneous *L. amazonensis* model.^14,26,27^ These findings provide proof-of-concept activity at a single dose; however, additional dose–response studies will be required to define potency and optimal dosing parameters.

### Li1 Does Not Induce Hepatic or Renal Toxicity *In Vivo* in a Prophylactic Model

Plasma biochemical analyses were performed to monitor systemic hepatic and renal parameters in BALB/c mice infected with *L. amazonensis* preincubated with the peptides Li1 or Li1nc (0.2 mg mL^–1^, 15 min). The evaluated biomarkers included alkaline phosphatase (ALP), alanine aminotransferase (ALT), aspartate aminotransferase (AST), and total, direct, and indirect bilirubin for hepatic function, as well as creatinine and urea for renal function (Figure 10).

**Figure 10:**
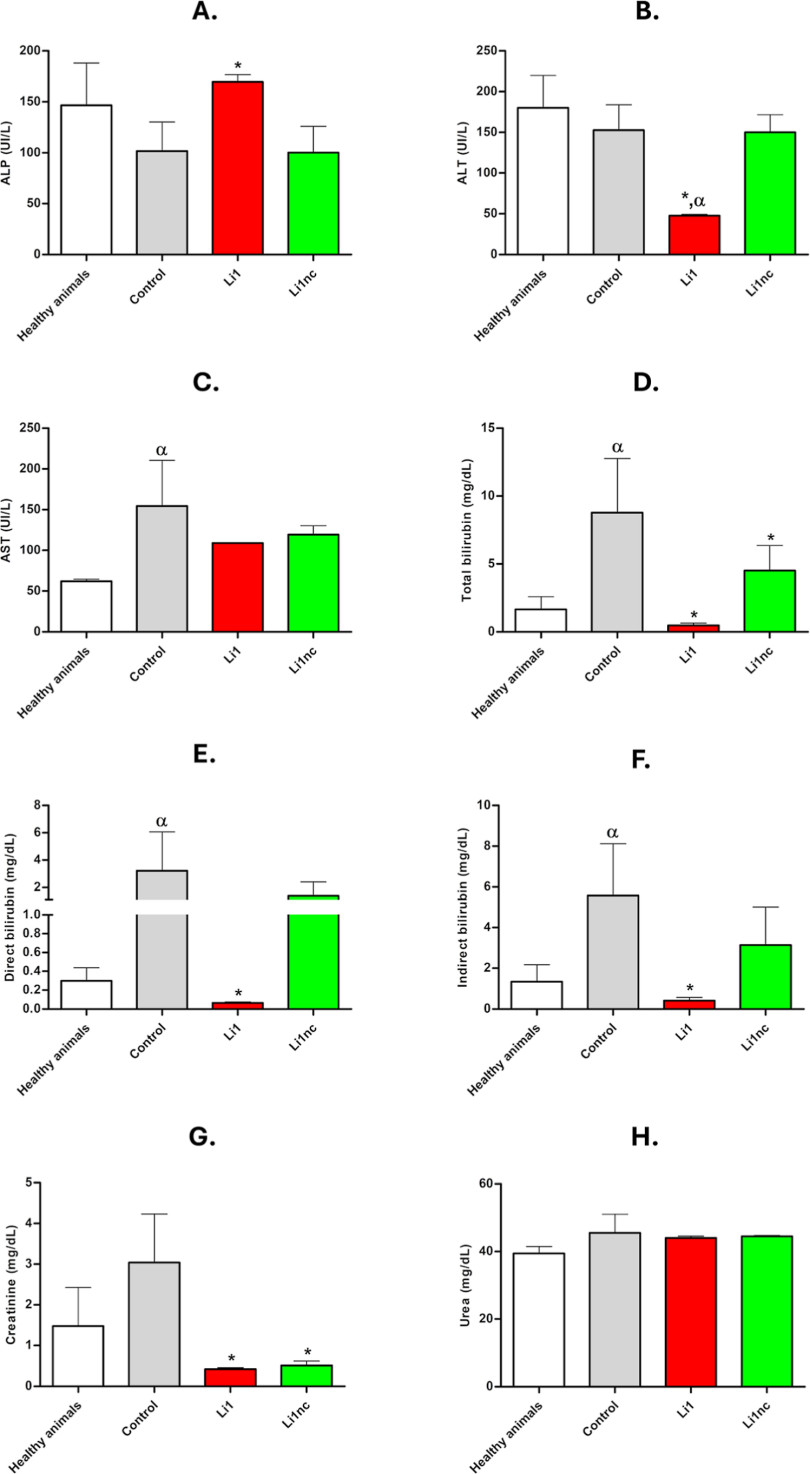
Plasma levels of hepatic and renal biochemical markers in BALB/c mice infected with *L. amazonensis* preincubated with Li1 or Li1nc peptides. (A) Alkaline phosphatase (ALP); (B) alanine aminotransferase (ALT); (C) aspartate aminotransferase (AST); (D–F) total, direct, and indirect bilirubin; (G) creatinine; and (H) urea. Mice were infected in the right hind paw with *L. amazonensis* metacyclic promastigotes pre-exposed to each peptide (0.2 mg mL^–1^, 15 min). Plasma samples were collected 30 days postinfection for biochemical evaluation. Data are expressed as mean ± standard deviation (*n* = 5 per group). Asterisks (*) denote statistically significant differences compared with the infected (untreated) group (*P* < 0.05); and Greek letters (α) indicate significant differences compared with the healthy group (*P* < 0.05).

No significant differences were observed between the Li1- or Li1nc-treated groups and healthy controls for any hepatic or renal parameter, except for a slight decrease in ALT levels in the Li1 group, which is not clinically relevant for indicating hepatocellular injury.^28,29^ In contrast, infected untreated mice showed significantly higher AST and ALT levels and increased total, direct, and indirect bilirubin compared with healthy animals, consistent with mild hepatic dysfunction and cholestasis typically associated with *L. amazonensis* infection.^30–32^ These elevations were not observed in animals infected with parasites preincubated with Li1 or Li1nc.

Regarding renal function, creatinine levels were slightly elevated only in the infected control group, while remaining within normal ranges for Li1- and Li1nc-treated groups. Urea concentrations did not differ significantly among the groups.^33^

Taken together, these data indicate that preincubation of metacyclic promastigotes with Li1 or Li1nc prior to inoculation did not induce systemic biochemical alterations in the host, suggesting the absence of hepatic or renal toxicity under the experimental conditions. The maintenance of normal hepatic and renal markers supports the biosafety of the peptides and confirms that the reduction in parasite burden observed *in vivo* is not associated with off-target toxic effects.

### The Peptides are Not Effective *In Vivo* in a Treatment Model after Infection Has Been Established

The limiting dilution assay demonstrated that the Li1 and Li1nc peptides (36 mg Kg^1–^) did not reduce parasite burden (Figure 11A) when administered in an *in vivo* postinfection treatment model. As expected, only the positive control, the standard drug amphotericin B at 2 mg Kg^1–^, showed efficacy, resulting in a 43.9% reduction in parasite load. Notably, this group also exhibited a significant reduction in paw thickness (Figure 11B). Although they did not exhibit activity against intracellular forms (amastigotes), interestingly, the peptides showed no hepatotoxicity or nephrotoxicity (Figure S1, Supporting Information).

**Figure 11:**
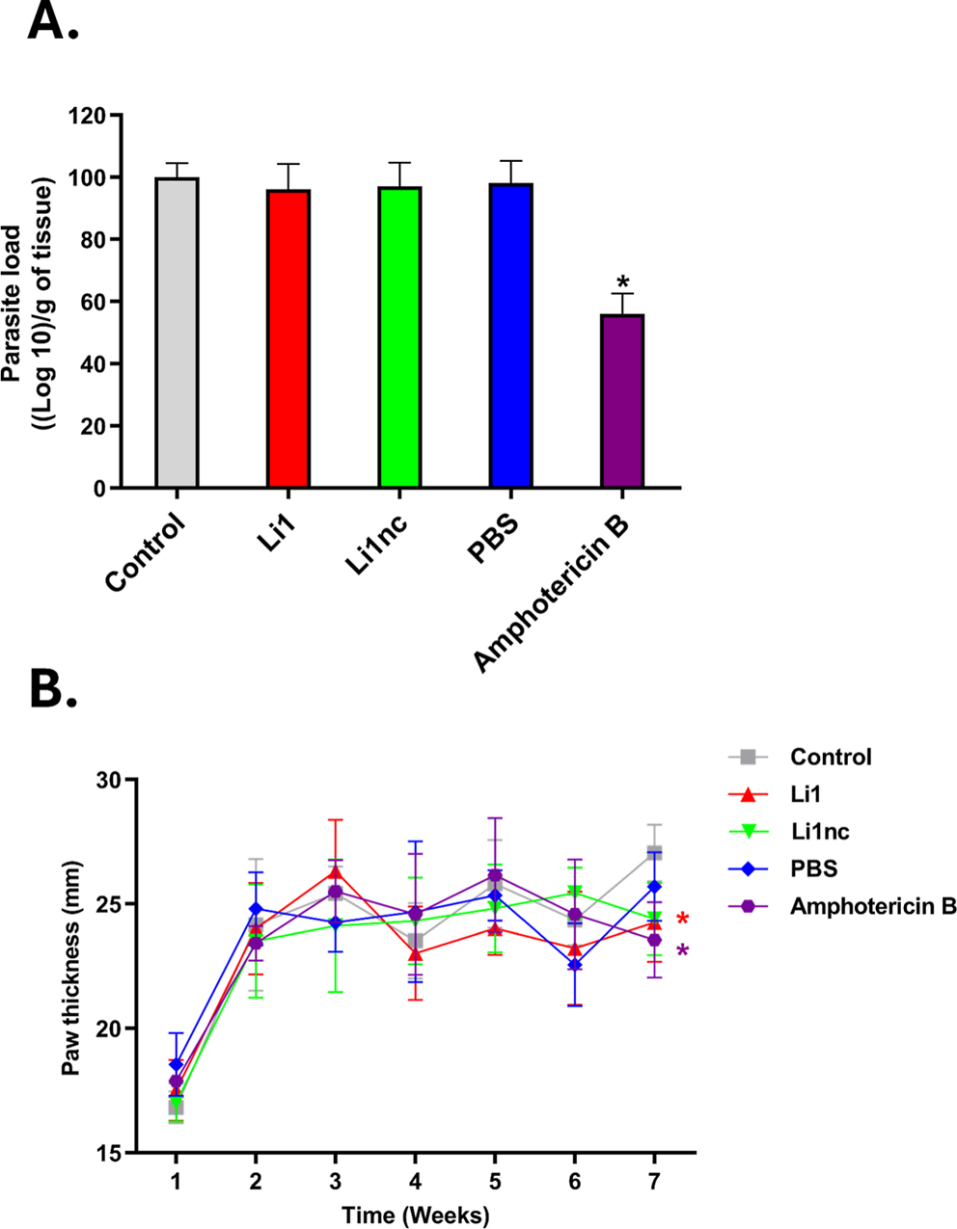
Parasite burden and lesion development in BALB/c mice infected with *L. amazonensis* promastigotes treated with Li1 or Li1nc peptides. (A) After 45 days of infection, treatment was administered for 1 week, and parasite load was determined by the limiting dilution method. (B) Paw thickness was measured weekly postinfection using a digital caliper. Stationary-phase promastigotes on day 6 of culture were inoculated into the right hind paw of BALB/c mice (*n* = 5 per group). After 45 days of infection, treatment was carried out at a dose of 36 mg Kg^–1^ for Li1 and Li1nc and 2 mg Kg^–1^ for Amphotericin B. The PBS group was included as the vehicle control. Data are expressed as mean ± standard deviation. Statistical analysis was performed by One-way ANOVA followed by Tukey’s post hoc test (*P* < 0.05). Asterisks (*) denote statistically significant differences compared with the control group (*P* < 0.05).

These *in vivo* findings demonstrate the lack of anti-*Leishmania* activity in a therapeutic setting, indicating that the peptides are not suitable as drugs for the treatment of established infection. In contrast, previous experiments involving preincubation of metacyclic promastigotes with the peptides resulted in reduced host cell invasion and, consequently, a lower parasite burden both *in vitro* and *in vivo*. This highlights a clear prophylactic potential, whereby the peptides act at the earliest stages of infection by interfering with parasite–host interactions before internalization.

Accordingly, the data support the use of the cyclic peptide as a prophylactic or infection-blocking agent rather than as a therapeutic compound. This distinction between prophylactic efficacy and the lack of therapeutic activity was not addressed in Verga et al.,^14^ and the present findings clarify that Li1 acts exclusively during the extracellular phase of infection, prior to parasite entry into host cells.

Although a cyclic scrambled control peptide could further refine the mechanistic interpretation, the present results, together with the lack of activity of the scrambled analogue reported by Verga et al.,^14^ consistently support a structure-dependent effect involving both sequence and conformational constraints. In this study, the term “cyclization” is used in the functional sense (cysteine-mediated disulfide constraint), and no advanced structural analysis was performed. Although our results demonstrate a functional impact of cyclization, detailed structural characterization and evaluation of additional structural controls (e.g., cyclic scrambled analogues or cysteine-blocked variants) will be pursued in future studies.

## Conclusion

The cyclic peptide Li1, identified by phage display for its affinity for *Leishmania* surface molecules, exhibited a reproducible ability to reduce parasite internalization and burden of *L. amazonensis* both *in vitro* and *in vivo*. Comparative analyses with its linear analogue, Li1nc, highlight the critical contribution of peptide cyclization to biological activity, conferring structural stability and enhanced interaction with the parasite surface. These findings corroborate that, in addition to the arrangement of the remaining amino acid residues, as shown by Verga et al.,^14^ cyclization mediated by the cysteines at positions 2 and 11 also contributes to Li1 activity. Pre-exposure of infective forms to Li1 led to a significant decrease in infection rates in macrophage models and to a reduced parasite load in a murine model of cutaneous leishmaniasis, without inducing hepatic or renal alterations detectable in systemic biochemical assays. In contrast, in a postinfection treatment model, after *in vivo* infection had been established, the peptides exhibited no effect on parasite burden, indicating a lack of activity once the intracellular cycle is established. Collectively, these findings indicate that the inhibitory effect of Li1 is structure-dependent and is not associated with host toxicity under the conditions evaluated.

However, the comparison between Li1 and Li1nc must be interpreted with caution, as the Cys → Ser substitution in the linear analogue introduces differences in side-chain chemistry (thiol versus hydroxyl), hydrogen-bonding capacity, and local polarity. Thus, the reduced activity of Li1nc cannot be attributed exclusively to the loss of cyclization and may reflect a combination of conformational and side-chain effects.

Furthermore, cyclic scrambled controls evaluated by Verga et al.^14^ were reported to be inactive, providing important mechanistic support for the findings observed in the present study. A scrambled version of the Li1 peptide (Li1scr, sequence: ACRKNRESNACP),^14^ which retains the same amino acid composition as Li1 but has a randomized sequence, exhibited no activity in either *in vitro* macrophage internalization assays or *in vivo* *L. infantum* infection models. These results establish the sequence dependent nature of Li1 activity and strongly support the specificity of the cyclic peptide interaction observed here. In this context, the biological effects reported in the present work are consistent with a mechanism that relies on defined sequence motifs rather than nonspecific, structure dependent surface interference, reinforcing the interpretation of Li1 as a targeted and specific peptide based prophylactic agent.

Although the precise molecular targets of Li1 remain to be identified, the present data consistently support a structure-dependent disruption of early parasite–host interactions, without allowing conclusions about sequence-specific receptor engagement. Li1 demonstrated activity at the tested dose, establishing initial evidence of *in vivo* efficacy; however, the pharmacological profile, including dose dependency, remains to be defined. The peptide’s selective interference with parasite internalization and the absence of systemic toxicity suggest that it represents a safe and rational scaffold for the development of peptide-based prophylactic approaches. The translational relevance of Li1 lies in its potential use for infection-blocking or paratransgenic strategies aimed at disrupting the transmission of metacyclic *Leishmania* forms within the vector and vertebrate hosts.

## Materials and Methods

Detailed Materials and Methods are available in the Supporting Information.

## Supplementary Material



## Notes

All materials and methods are described in detail in the Electronic Supporting Information (ESI). Data on macrophage and promastigote cell viability are provided in Table S1, whereas hepatotoxicity and nephrotoxicity profiles from the postinfection treatment model are presented in Figure S1 (ESI) (PDF)

## Abbreviations

SPPS: solid-phase peptide synthesis
THP-1: Human monocytic cell line derived from an acute monocytic leukemia patient
CC_50_: 50% cytotoxic concentration
IC_50_: 50% inhibitory concentration
NTDs: neglected tropical diseases
WHO: World Health Organization
LPG: lipophosphoglycan
GP63: glycoprotein 63
PPG: proteophosphoglycan
KMP-11: kinetoplastid membrane protein 11
GP46: glycoprotein 46
HASP: hydrophilic acylated surface protein
HOBt: hydroxybenzotriazole
DIC: diisopropylcarbodiimide
DMF: dimethylformamide
RP-HPLC: reverse-phase high-performance liquid chromatography
TFA: trifluoroacetic acid
ESI-IT-MS: electrospray ionization-ion trap-mass spectrometry
LIT: liver infusion tryptose
RPMI: Roswell Park Memorial Institute
FBS: fetal bovine serum
BOD: biological oxygen demand (incubator)
LM: light microscopy
PBS: phosphate-buffered saline
PMA: phorbol 12-myristate 13-acetate
MTT: 3-(4,5-dimethylthiazol-2-yl)-2,5-diphenyltetrazolium bromide
PMS: phenazine methosulfate
BSA: bovine serum albumin
AST: aspartate aminotransferase
ALT: alanine aminotransferase
ALP: alkaline phosphatase
SBCAL: Sociedade Brasileira de Ciência de Animais de Laboratório
CONCEA: conselho nacional de controle da experimentação animal
ANOVA: analysis of variance
MM: molecular mass
*m*/*z*: mass-to-charge ratio
Z: charge state
ROS: reactive oxygen species
CR1: complement receptor 1
CR3: complement receptor 3
TLR2: toll-like receptor 2
TLR4: toll-like receptor 4
