# Supplementary material for: Functional Effects of Cyclization on Li1 Peptide Activity against Metacyclic Leishmania amazonensis Internalization
Source: ACS Omega. 2026 Mar 2;11(10):16164–76. doi: 10.1021/acsomega.5c11292 (PMC13000623; doi:10.1021/acsomega.5c11292)
Supplement: Supplementary file 1 [file ao5c11292_si_001.pdf]

Supplementary Information

Functional Effects of Cyclization on Li1 Peptide Activity Against Metacyclic *Leishmania amazonensis* Internalization

*Túlio Custódio Reis[1,†], Ana Clara Lunardi Yagi[1,†], Angela Maria Arenas Velásquez[1,2], Ana Laura Dias Ramos[1], Natália Caroline Costa Coelho[1], Eduardo Maffud Cilli[3], Márcia A. S. Graminha[1]**

[1]Department of Clinical Analysis, School of Pharmaceutical Science, São Paulo State University (UNESP), Araraquara 14800-903, Brazil

[2]Latin American Institute of Life and Natural Sciences, Federal University of Latin American Integration (UNILA), Foz do Iguaçu 85870-650, Brazil

[3]Department of Biochemistry and Chemical Technology, Institute of Chemistry, São Paulo State University (UNESP), Araraquara 14800-060, Brazil

[†]The authors contributed equally.

***Corresponding author:** Márcia A. S. Graminha, e-mail: marcia.graminha@unesp.br

MATERIALS AND METHODS

Peptide Synthesis and Characterization

The cyclic peptide Li1 (Sequence: Biotin-EC|KRARSAPNC|N-COOH) with an 8-amino-acid loop and linear Li1nc (Sequence: Biotin-ESKRARSAPNSN-COOH) peptide were synthesized manually by solid-phase peptide synthesis (SPPS) using Fmoc chemistry as previously described.[1,2] A Wang resin (0.1 mmol scale, 0.4 equiv.) was used to obtain peptides with a C-terminal carboxylic acid. Coupling reactions were performed with equimolar hydroxybenzotriazole (HOBt) and diisopropylcarbodiimide (DIC) for 2 h under constant agitation. N-terminal deprotection was achieved with 20% 4-methylpiperidine in dimethylformamide (DMF).[3,4] Biotin was coupled to the N-terminus under identical conditions. Cyclization of Li1 was carried out by oxidation with iodine (0.06 M in methanol) using the crude peptide (0.1 mg mL^{-1} in ultrapure water), followed by neutralization with ascorbic acid (1 M).[5,6]

Peptides were purified by semi-preparative reverse-phase high-performance liquid chromatography (RP-HPLC) using a C18 column (25 cm x 10 mm, 5 µm; Jupiter Proteo) with a linear gradient of 5–95% solvent B (0.036% TFA in acetonitrile) over 30 min at a flow rate of 5 mL min^{-1}. Elution was monitored at 220 nm. Analytical purity was confirmed on a C18 analytical column under identical gradient conditions. Molecular masses were verified by electrospray ionization–ion trap mass spectrometry (ESI-IT-MS; LCQ Fleet, Thermo Fisher Scientific) in positive ion mode over an m/z range of 200–2000.

Parasite and Cell Cultures

L. amazonensis (strain MPRO/BR/1972/M1841-LV79) promastigotes were cultured in Liver Infusion Tryptose (LIT) medium (pH 7.5) supplemented with 13.4 µg mL^{-1} penicillin, 0.2 mg mL^{-1} streptomycin (Sigma), and 10% (v/v) heat-inactivated fetal bovine serum (FBS; Thermo) at 26°C.[7]

THP-1 cells (human monocytic cell line) were cultured in Roswell Park Memorial Institute (RPMI) 1640 medium supplemented with 10% FBS, penicillin, and streptomycin under a humidified atmosphere of 5% CO_2 at 37°C.[8] Cells were differentiated into macrophage-like forms with 100 nM phorbol 12-myristate 13-acetate (PMA) for 96 h before infection assays.

Growth Curve and Metacyclic Form Enrichment

Parasite growth was monitored for 8 days after inoculation (2 x 10^5 promastigotes mL^{-1}) by counting daily using a Neubauer chamber. The stationary phase was identified from day 5 onward. The proportion of metacyclic promastigotes was determined by Giemsa staining on days 5–7, based on morphological criteria (flagellum length ≥1x cell body).[9] Metacyclic forms were enriched from day 6–7 cultures by Ficoll density-gradient centrifugation (10–40%) at 1,300 *g* for 10 min at 4°C, following the protocol of Späth and Beverley.[9] Fractions enriched in metacyclic forms were collected for *in vitro* and *in vivo* assays.

Isolation of Murine Peritoneal Macrophages

Resident peritoneal macrophages were obtained from male Swiss mice (4–8 weeks old, 25 g) injected intraperitoneally with 1 mL of 3% sodium thioglycolate solution (Difco). After 3–4 days, animals were euthanized according to ethical guidelines, and the peritoneal exudate was collected in sterile PBS. Cells were used in cytotoxicity and infection assays.[10]

***In vitro* Infection Assays**

Differentiated THP-1 macrophages and murine peritoneal macrophages (2 x 10^4 cells per well) were seeded on 13 mm glass coverslips in 24-well plates. Enriched *L. amazonensis* metacyclic promastigotes (1 x 10^5) were pre-exposed to Li1 or Li1nc peptides (0.5 mg mL^{-1}, is equivalent to 0.318 mmol L^{-1} (Li1), and 0.324 mmol L^{-1} (Li1nc); 15 min, 26°C) and then co-incubated with macrophages for 2 h at 37°C and 5% CO_2. After infection, cells were fixed with methanol, stained with Giemsa, and examined by light microscopy (1000x). The infection rate (% infected macrophages) and infection index (mean parasites per macrophage x % infected cells) were

determined by counting 100 macrophages per sample. Statistical comparisons were performed relative to untreated controls.[10,11]

Antipromastigote and Cytotoxicity Assays

The antipromastigote activity of the peptides was determined by the MTT [3-(4,5-dimethylthiazol-2-yl)-2,5-diphenyltetrazolium bromide] assay. Stationary-phase promastigotes (1 x 10^7 promastigotes mL^{-1}) were incubated with peptides (0.5 mg mL^{-1}, 72 h, 26°C). Amphotericin B (0.19–12.5 µg mL^{-1}) served as a reference. Absorbance was measured at 570 nm (Tecan), and IC_{50} values were determined by polynomial regression.[10] Cytotoxicity toward THP-1 and murine macrophages was assessed by the MTT assay after 24 h exposure to peptides (0.5 mg mL^{-1}, 37°C). Amphotericin B (4.68–300 µg mL^{-1}) was used as a positive control. CC_{50} values were calculated by quadratic regression.[10]

Confocal Fluorescence Microscopy

Localization of peptides on parasite and host cell surfaces was evaluated by confocal microscopy. Fixed *L. amazonensis* metacyclics (1 x 10^5 promastigotes mL^{-1}), THP-1–derived macrophages, or murine peritoneal macrophages (2 x 10^4 cells mL^{-1}) were blocked with 4% (v/v) bovine serum albumin (BSA) and incubated with biotinylated Li1 or Li1nc peptides (0.5 mg mL^{-1}, 8 h). After washing, samples were incubated with Alexa Fluor 488–conjugated streptavidin for 1 h and counterstained with Hoechst 33342. Coverslips were mounted with Corning Cell–Tak adhesive and imaged using a Zeiss LSM800 confocal microscope under identical laser and gain settings. A PBS + streptavidin control was included to verify labeling specificity.[11] For fluorescence quantification, the method described by Shihan et al.[12] was employed using the Fiji (ImageJ) software. The adopted approach consisted of measuring the mean fluorescence intensity (MFI) within a region of interest (ROI) on the cell surface, followed by background subtraction, using independent replicates.

Prophylactic Model of *in vivo* Infection Assay

Male BALB/c mice (20–30 g; n = 5 per group) were divided into four groups: healthy, infected control, Li1, and Li1nc. Stationary-phase promastigotes (1 x 10^7 promastigotes mL^{-1}) were pre-exposed to Li1 or Li1nc (0.2 mg mL^{-1}, is equivalent to 0.127 mmol L^{-1} (Li1) and 0.130 mmol L^{-1} (Li1nc); 15 min, 26°C), before inoculation (100 µL) into the right hind paw. Animals were monitored for 30 days, and paw thickness was measured weekly using a digital caliper. At the endpoint, parasite burden was determined by limiting dilution of paw tissue homogenates in complete LIT medium. Cultures were incubated at 26°C and monitored daily for promastigotes growth. Parasite burden was expressed as relative and percentage reductions versus control.[11,10,13]

Biochemical Analyses of Hepatic and Renal Function in a Prophylactic *In Vivo* Model

At euthanasia (day 30), blood samples were collected by cardiac puncture, and plasma was analyzed for hepatic (ALP, ALT, AST, total/direct/indirect bilirubin) and renal (urea, creatinine) biomarkers using standard colorimetric and enzymatic assays. Analyses were performed at the São José Veterinary Laboratory (Matão, São Paulo, Brazil).[14-20]

Therapeutic *in vivo* Treatment Model After Infection Establishment

Male BALB/c mice (20–30 g; n = 5 per group) were allocated into six groups: healthy, infected control, Li1, Li1nc, PBS, and amphotericin B (positive control). In the infected groups, stationary-phase promastigotes (1 x 10^7 promastigotes mL^{-1}) were inoculated (100 µL) into the right hind paw. Animals were monitored for 45 days, and paw thickness was measured weekly using a digital caliper. After this period, treatment (50 µL) was initiated and administered for 1 week via intradermal injection directly into the dorsum of the infected paw. For the calculation of the dose for the Li1 and Li1nc groups, the base concentration of 0.2 mg mL^{-1} used in the prophylactic *in vivo* assay was employed,[11,5] resulting in a daily dose of 36 mg Kg^{-1}, administered in three divided doses (12 mg Kg^{-1}) every 8 h. The amphotericin B group received a dose of 2 mg Kg^{-1}, as previously established by Velásquez et al.[10] At the end of the experiment, parasite burden was

determined by limiting dilution of paw tissue homogenates in complete LIT medium. Cultures were incubated at 26 °C and monitored daily for promastigotes growth. Parasite burden was expressed as relative and percentage reductions compared with the infected control.[10]

Ethical Statement

All animal procedures were conducted following Brazilian guidelines (SBCAL/CONCEA) and approved by the Ethics Committee for Animal Experimentation of the São Paulo State University (CEUA/FCF/Car nº 6499092515).

Data Analysis

All experiments were performed in independent triplicates or with five animals per group for *in vivo* assays. Results are expressed as mean ± standard deviation (SD). Statistical analyses were conducted using BioEstat 5.3 and GraphPad Prism 8.0 software. One-way ANOVA followed by Tukey's post hoc test was applied for multiple comparisons, and the unpaired two-tailed Student's t-test was used for pairwise comparisons. The value of $P < 0.05$ was considered statistically significant.

RESULTS

Table S1. Cell viability percentages (%) of macrophage models and *L. amazonensis* following exposure to Li1 and Li1nc peptides at a concentration of 0.5 mg/mL

**Treatment**	**Cell viability (%) (Mean ± SD)**		
	Murine macrophage	THP-1 macrophage	**L. amazonensis*
Li1	107.5 ± 5.2	101.8 ± 4.5	110.3 ± 8.0
Li1nc	113.3 ± 1.6	92.1 ± 12.4	94.0 ± 4.0
#No treatment	100 ± 1.3	100 ± 7.7	100.9 ± 0.4

* Stationary-phase promastigotes; # No treatment = murine and THP-1 macrophages or stationary-phase *L. amazonensis* promastigotes not exposed to the peptides; SD=Standard deviation.

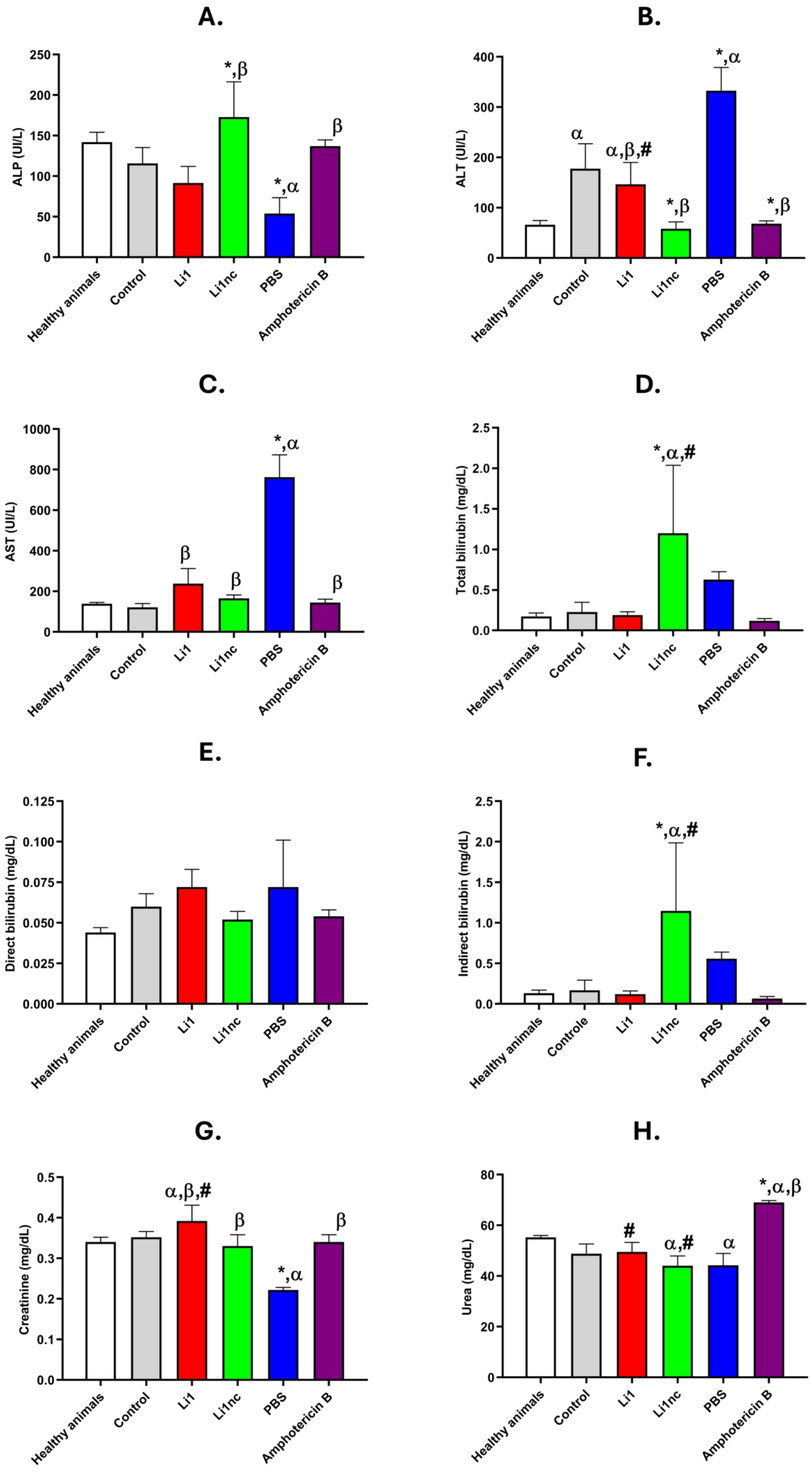

A.
ALP (UI/L)
*,β
β
*,α
B.
ALT (UI/L)
*,α
α
α,β,#
*,β
*,β
C.
AST (UI/L)
*,α
β
β
β
D.
Total bilirubin (mg/dL)
*,α,#
E.
Direct bilirubin (mg/dL)
F.
Indirect bilirubin (mg/dL)
*,α,#
G.
Creatinine (mg/dL)
α,β,#
β
β
*,α
H.
Urea (mg/dL)
*,α,β
#
α,#
α
Healthy animals
Control
Controle
Li1
Li1nc
PBS
Amphotericin B

Figure S1. Plasma levels of hepatic and renal biochemical markers in BALB/c mice infected with *Leishmania amazonensis* and treated with the Li1 or Li1nc peptides. (A) Alkaline phosphatase (ALP); (B) alanine aminotransferase (ALT); (C) aspartate aminotransferase (AST); (D–F) total, direct, and indirect bilirubin; (G) creatinine; and (H) urea. After 45 days of infection, mice were subsequently treated for one week with a daily dose of 36 mg Kg^{-1} of the peptides and 2 mg Kg^{-1} of amphotericin B. Plasma samples were collected after treatment for biochemical evaluation. Data are expressed as mean ± standard deviation (n = 5 per group). Asterisks (*) indicate statistically significant differences compared with the untreated control group ($P < 0.05$); Greek letters (α and β) indicate significant differences ($P < 0.05$) compared with the healthy and PBS groups, respectively; and hashtags (#) indicate significant differences ($P < 0.05$) compared with the amphotericin B group.

REFERENCES

(1) Merrifield, R. B. Solid-Phase Peptide Synthesis. III. An Improved Synthesis of Bradykinin. Biochemistry 1964, 3 (9), 1385–1390. https://doi.org/10.1021/bi00897a032

(2) Bonnycastle, L. L. C.; Mehroke, J. S.; Rashed, M.; Gong, X.; Scott, J. K. Probing the Basis of Antibody Reactivity with a Panel of Constrained Peptide Libraries Displayed by Filamentous Phage. J. Mol. Biol. 1996, 258 (5), 747–762. https://doi.org/10.1006/jmbi.1996.0284

(3) Costa, N. C. S.; Dos Anjos, L. R.; de Souza, J. V. M.; de Arruda Brasil, M. C. O.; Moreira, V. P.; Graminha, M. A. S.; Lubec, G.; Gonzalez, E. R. P.; Cilli, E. M. Development of New Leishmanicidal Compounds via Bioconjugation of Antimicrobial Peptides and Antileishmanial Guanidines. ACS Omega 2023, 8, 34008–34016. https://doi.org/10.1021/acsomega.3c04878.

(4) Coelho, N. C. S.; Portuondo, D. L. F.; Lima, J.; Velásquez, A. M. A.; Valente, V.; Carlos, I. Z.; Cilli, E. M.; Graminha, M. A. S. Peptide Dimerization as a Strategy for the Development of Antileishmanial Compounds. Molecules 2024, 29, 5170. https://doi.org/10.3390/molecules29215170.

(5) Reis, T. C. Síntese do peptídeo Li1 e sua contribuição para a inibição da internalização de formas infectantes de Leishmania amazonensis em macrófagos: estudos in vitro e in vivo em modelo de leishmaniose cutânea. Dissertação de Mestrado, Universidade Estadual Paulista (Unesp), Araraquara, 2025. https://hdl.handle.net/11449/312288

(6) Reddy, K. M. B.; Kumari, Y. B.; Mallikharjunasarma, D.; Bulliraju, K.; Sreelatha, V.; Ananda, K. Large Scale Solid Phase Synthesis of Peptide Drugs: Use of Commercial Anion Exchange Resin as Quenching Agent for Removal of Iodine during Disulphide Bond Formation. Int J Pept 2012, 2012. https://doi.org/10.1155/2012/323907

(7) Fernandes, J. F.; Castellano, O. Growth Characteristics and Chemical Composition of Trypanosoma cruzi. Exp. Parasitol. 1966, 18 (2), 1–9. https://doi.org/10.1016/0014-4894(66)90016-6

(8) Gogulamudi, V. R.; Dubey, M. L.; Kaul, D.; Atluri, V. S. R.; Sehgal, R. Downregulation of Host Tryptophan-Aspartate Containing Coat (TACO) Gene Restricts the Entry and Survival of Leishmania donovani in Human Macrophage Model. Front. Microbiol. 2015, 6, 946. https://doi.org/10.3389/fmicb.2015.00946

(9) Späth, G. F.; Beverley, S. M. A Lipophosphoglycan-Independent Method for Isolation of Infective Leishmania Metacyclic Promastigotes by Density Gradient Centrifugation. Exp Parasitol 2001, 99 (2), 97–103. https://doi.org/10.1006/expr.2001.4656.

(10) Velásquez, A. M. A.; Ribeiro, W. C.; Venn, V.; Castelli, S.; Santoro de Camargo, M.; Pires de Assis, R.; Alves de Souza, R.; Rimoldi Ribeiro, A.; Gaban Passalacqua, T.; Aristeu da Rosa, J.; Martins Baviera, A.; Mauro, A. E.; Desideri, A.; Almeida-Amaral, E. E.; Graminha, M. A. S. Efficacy of a Binuclear Cyclopalladated Compound Therapy for Cutaneous Leishmaniasis in the Murine Model of Infection with Leishmania amazonensis and Its Inhibitory Effect on Topoisomerase 1B. Antimicrob. Agents Chemother. 2017, 61 (e00688-17). https://doi.org/10.1128/AAC.00688-17.

(11) Verga, J. B. M.; Graminha, M. A. S.; Jacobs-Lorena, M.; Cha, S. J. Peptide Selection via Phage Display to Inhibit *Leishmania*-Macrophage Interactions. Front Microbiol 2024, 15. https://doi.org/10.3389/fmicb.2024.1362252.

(12) Shihan, M. H.; Novo, S. G.; Le Marchand, S. J.; Wang, Y.; Duncan, M. K. A simple method for quantitating confocal fluorescent images. Biochem. Biophys. Rep. 25 (2021) 100916. https://doi.org/10.1016/j.bbrep.2021.100916.

(13) TITUS, R. G.; MARCHAND, M.; BOON, T.; LOUIS, J. A. A Limiting Dilution Assay for Quantifying Leishmania major in Tissues of Infected Mice. Parasite Immunol 1985, 7 (5), 545–555. https://doi.org/10.1111/j.1365-3024.1985.tb00098.x.

(14) Moreira, V. P.; da Silva Mela, M. F.; Anjos, L. R. dos; Saraiva, L. F.; Arenas Velásquez, A. M.; Kalaba, P.; Fabisiková, A.; Clementino, L. da C.; Aufy, M.; Studenik, C.; Gajic, N.; Prado-Roller, A.; Magalhães, A.; Zehl, M.; Figueiredo, I. D.; Baviera, A. M.; Cilli, E. M.; Graminha, M. A. S.; Lubec, G.; Gonzalez, E. R. P. Novel Selective and Low-Toxic Inhibitor of LmCPB2.8ΔCTE (CPB) One Important Cysteine Protease for Leishmania Virulence. Biomolecules 2022, 12 (12). https://doi.org/10.3390/biom12121903.

(15) Godoy, P.; Salles, P. G. O. Associação de leishmaniose visceral e hepatite B de curso fulminante: relato de um caso. Rev. Soc. Bras. Med. Trop. 2002, 35 (5), 515–518. https://doi.org/10.1590/S0037-86822002000500015

(16) Rigo, R. S.; Rigo, L.; Honer, M. R. Aspectos Clínicos e Laboratoriais na Leishmaniose Visceral Americana. J. Bras. Nefrol. 2009, 31 (1), 48–54.

(17) Fernandes, J. F.; Castellano, O. Growth Characteristics and Chemical Composition of Trypanosoma cruzi. Exp. Parasitol. 1966, 18 (2), 1–9. https://doi.org/10.1016/0014-4894(66)90016-6

(18) Gogulamudi, V. R.; Dubey, M. L.; Kaul, D.; Atluri, V. S. R.; Sehgal, R. Downregulation of Host Tryptophan-Aspartate Containing Coat (TACO) Gene Restricts the Entry and Survival of Leishmania donovani in Human Macrophage Model. Front. Microbiol. 2015, 6, 946. https://doi.org/10.3389/fmicb.2015.00946

(19) TITUS, R. G.; MARCHAND, M.; BOON, T.; LOUIS, J. A. A Limiting Dilution Assay for Quantifying Leishmania major in Tissues of Infected Mice. Parasite Immunol 1985, 7 (5), 545–555. https://doi.org/10.1111/j.1365-3024.1985.tb00098.x.

(20) do Espírito Santo, R. D.; Velásquez, Á. M. A.; Passianoto, L. V. G.; Sepulveda, A. A. L.; da Costa Clementino, L.; Assis, R. P.; Baviera, A. M.; Kalaba, P.; dos Santos, F. N.; Éberlin, M. N.; da Silva, G. V. J.; Zehl, M.; Lubec, G.; Graminha, M. A. S.; González, E. R. P. N, N′ N″-Trisubstituted Guanidines: Synthesis, Characterization and Evaluation of Their Leishmanicidal Activity. Eur J Med Chem 2019, 171, 116–128. https://doi.org/10.1016/j.ejmech.2019.03.032.
